# Fibroblasts of Machado Joseph Disease patients reveal autophagy impairment

**DOI:** 10.1038/srep28220

**Published:** 2016-06-22

**Authors:** Isabel Onofre, Nuno Mendonça, Sara Lopes, Rui Nobre, Joana Barbosa de Melo, Isabel Marques Carreira, Cristina Januário, António Freire Gonçalves, Luis Pereira de Almeida

**Affiliations:** 1 CNC-Center for Neuroscience and Cell Biology, Coimbra, Portugal; 2 Faculty of Pharmacy, University of Coimbra, Coimbra, Portugal; 3 Neurology Department, Coimbra University Hospital Center, Coimbra, Portugal; 4 Faculty of Medicine, University of Coimbra, Portugal; 5 IIIUC- Institute for Interdisciplinary Research, University of Coimbra, Coimbra, Portugal; 6 Cytogenetics and Genomics Laboratory, Faculty of Medicine, University of Coimbra, Coimbra, Portugal

## Abstract

Machado Joseph Disease (MJD) is the most frequent autosomal dominantly inherited cerebellar ataxia caused by the over-repetition of a CAG trinucleotide in the *ATXN3* gene. This expansion translates into a polyglutamine tract within the ataxin-3 protein that confers a toxic gain-of-function to the mutant protein ataxin-3, contributing to protein misfolding and intracellular accumulation of aggregates and neuronal degeneration. Autophagy impairment has been shown to be one of the mechanisms that contribute for the MJD phenotype. Here we investigated whether this phenotype was present in patient-derived fibroblasts, a common somatic cell type used in the derivation of induced pluripotent stem cells and subsequent differentiation into neurons, for *in vitro* disease modeling. We generated and studied adult dermal fibroblasts from 5 MJD patients and 4 healthy individuals and we found that early passage MJD fibroblasts exhibited autophagy impairment with an underlying mechanism of decreased autophagosome production. The overexpression of beclin-1 on MJD fibroblasts reverted partially autophagy impairment by increasing the autophagic flux but failed to increase the levels of autophagosome production. Overall, our results provide a well-characterized MJD fibroblast resource for neurodegenerative disease research and contribute for the understanding of mutant ataxin-3 biology and its molecular consequences.

Machado Joseph Disease (MJD) also known as Spinocerebellar Ataxia Type 3 (SCA3) is an autosomal dominant inherited cerebellar ataxia and a progressive, adult-onset neurodegenerative disease^1^,^2^. SCA3 is caused by a CAG-repeat expansion in the *ATXN3* gene on chromosome 14q24.3–q32.2, which results in an abnormally long polyglutamine tract in the ataxin-3 protein^3^.

There is strong evidence that proteins with an overlong mutant polyglutamine tract are inefficiently degraded by the ubiquitin-proteosome system (UPS) but may be cleared by macroautophagy (hereafter referred to as autophagy), an intracellular degradation pathway with a crucial role in degradation of insoluble aggregate-prone proteins^4^ such as the polyglutamine proteins involved in neurodegenerative diseases^5^. Our group previously provided evidence of an impairment of the autophagy pathway in a MJD rodent model and decreased levels of Beclin-1/ATG6, a component of the class III PI3 kinase complex required for autophagy initiation and autophagosome formation, in human fibroblasts from two MJD patients^6^.

Adult dermal fibroblasts are an accessible source of patient cells, easy to grow in culture and currently the most suitable somatic cell type for reprogramming giving an efficient yield of induced pluripotent stem cells (iPSCs)^7^. Studying patient-derived fibroblasts, as somatic cell type of origin can give new insights in the establishment of diseased phenotype of patient-derived neurons resulting from iPSCs differentiation, taking in account that fibroblasts hold the native genetic background of the patient without further genetic manipulation^8^,^9^.

Therefore, in this work we collected and studied a cohort of human primary fibroblast cultures obtained from MJD patients and healthy controls in order to elucidate whether this type of peripheral cells presents a MJD related phenotype, at molecular, cellular or functional level. For this purpose, we examined the levels of i) Beclin-1/ATG6 and ii) p62/SQSTM, a protein with an ubiquitin-associated domain that is involved in interaction with ubiquitinated proteins and transport to autophagosomes; p62/SQSTM interacts with LC3, a protein present in autophagic membranes, through its LC3 recognition sequence (LRS) allowing the incorporation of ubiquitinated proteins in autophagosomes to be subsequently degraded in autolysosomes^10^. To clarify whether a) autophagosome formation is impaired or b) the autophagic flux (rate of autophagosome delivery to lysosomes) is compromised we investigated the levels of LC3-II^11^.

We used primary skin fibroblasts as an extraneural disease model to study the underlying mechanism of molecular autophagic dysfunction associated to MJD. This strategy has been used for other neurodegenerative disorders such as Parkinson’s disease, Huntington’s disease and Alzheimer’s disease^12^,^13^,^14^ to complement studies in animal models, transformed cell lines and patient tissues. Therefore, in this work we aimed at evaluating the MJD phenotype in human adult fibroblasts and to further use it as starting material for reprogramming and implementation of disease models.

## Materials and Methods

### Cell culture

MJD and control fibroblasts were generated from 3 mm forearm and thigh dermal biopsies following informed consent under protocols approved by the Hospital Center of the University of Coimbra and the Medical Faculty of the University of Coimbra and in accordance with the guidelines stipulated by the hospital Ethics Committee. All the fibroblasts were cultured in complete culture medium (DMEM (Gibco), supplemented with 10% FBS (Gibco), 2 mM L-Glu (Gibco), 1% penicillin/streptomycin (Gibco) and 1% NEAA (Sigma-Aldrich))^15^,^16^.

Briefly, skin explants were washed in PBS and subcutaneous tissue was excised. Epidermis was removed either mechanically or enzymatically (0.05% dispase/PBS, 1 h at 37 °C) and resulting dermal samples were cut in small pieces and placed in 0.1% gelatin coated tissue culture dishes.

Fibroblasts outgrowths were detected within a week and allowed to grow upon confluency in complete culture medium. Human fibroblasts were harvested with trypsin 0.05% and transferred to culture flasks for further expansion. Cell subculture was done when confluence was reached and using a 1:3 split ratio.

### Vector production and lentiviral transduction

The lentiviral vector encoding for human beclin-1 was produced as previously described^17^. Fibroblasts were incubated with virus for 12 h before changing culture medium and chloroquine treatments were performed 96 h after transduction.

### Karyotype analysis

The karyotype analysis was performed using standard G-banding techniques^18^. Cells cultured in a T25 flask were treated with 0.2 μg/ml Colcemid for up to 3 hours, followed by dissociation with trypsin/EDTA. The cells were pelleted via centrifugation and re-suspended in pre-warmed 0.05 M KCl hypotonic solution and incubated for 20 minutes. Following centrifugations, the cells were re-suspended in fixative. Metaphase spreads were prepared on glass microscope slides and GTG-banded by brief exposure to trypsin and stained with Giemsa. A minimum of 10 metaphase spreads were analysed for the fibroblasts. Karyotypes were established according to the International System for Human Cytogenetic Nomenclature (ISCN) 2013^19^.

### Immunocytochemistry

Cells were briefly fixed in a 50/50 mixture of ice cold acetone-methanol or PFA 4% for 10 min and then blocked in PBS containing 0.3% Triton X-100 and 5% FBS for 1 hr before incubation with primary antibodies (rabbit ataxin-3 1:1000 Immunostep, mouse ataxin-3, clone 1H9, 1:1000 Millipore, mouse TE-7 1:100 Millipore, mouse vimentin 1:100 Cell Signaling and rabbit LC3B 1:400 Cell Signaling) overnight at 4 °C, in PBS containing 0.3% Triton X-100 and 1% BSA. After three washes with PBS, cells were incubated with Alexa Fluor® 488 and 594 secondary antibodies (1:200, Invitrogen) for 2 h at room temperature. Additionally, cells were stained with DAPI in order to visualize cell nuclei and, after washing, mounted in Fluoroshield (Sigma-Aldrich Aldrich). Fluorescent signals were detected using a Zeiss inverted microscope (Zeiss Axio Observer Z1).

### Cell counts and quantification of ataxin-3 and LC3

LC3 and Ataxin-3 fluorescence were measured using a semiautomated image-analysis software package (Zen Observer, Germany). At least 30 cells for each condition were analyzed, using an x20 objective.

### Cell treatment

Fibroblasts were incubated with 100 μM chloroquine diphosphate (Sigma-Aldrich) dissolved in water in the treated group and with vehicle in non-treated group, during 4 h at 37 °C before protein extraction or imunnocytochemistry.

### Protein Isolation and Western Blot Analysis

The cells were lysed with RIPA buffer (50 mM Tris-Cl, pH 7.5, 150 mM NaCl, 1% Nonidet P-40, 0.5% sodium deoxycholate and 0.1% SDS, 1 mM PMSF and 1 mM DTT) supplemented with a protease inhibitor cocktail (Roche), triturated and centrifuged at 12,000 rpm for 20 min at 4 °C. The cell lysates were collected after centrifugation. The protein concentration in the lysates was determined using the Bradford protein assay reagent (Bio-Rad). Approximately 30 μg of protein was separated on SDS-PAGE gels (4% stacking and 10% or 12% running) and transferred to a PVDF membrane (Immobilon®-P Millipore). The blots were then incubated with primary antibodies against beclin-1 (1:1000, BD Biosciences), LC3B (1:1000, Cell Signaling), p62/SQSTM1 (1:1000, Cell Signaling), Ataxin-3 (clone 1H9 1:3000, Millipore) and actin (clone AC-74 1:5000, Sigma-Aldrich). As a control, membranes were re-probed for β-actin. The protein bands were visualized by using the corresponding alkaline phosphatase-linked secondary antibodies and ECF substrate (GE Healthcare) in a chemifluorescence device (VersaDoc Imaging System Model 3000, BioRad). For semiquantitative analysis, a partition ratio with actin or tubulin was calculated following quantification with Quantity-one 1-D image analysis software version 4.5.

### DNA extraction and genotyping

Genomic DNA was isolated from fibroblasts cultures using the Quick-gDNA Miniprep Genomic DNA Purification kit (Zymo® Research Corporation). All DNA samples were considered pure regarding the A260/A280 ratio that was comprised between1.8–2.0.

Two fragments of approximately 473 base pairs (bp) were generated by PCR reaction for allele 1 and allele 2, with customized primers for exon 10 of *ATXN3* gene. Those fragments were resolved in an agarose gel and further purified using the MiniElute Gel Extraction Kit (Qiagen).

A cloning reaction of the PCR fragments was performed with Zero Blunt® TOPO® PCR Cloning kit for Sequencing (Invitrogen) following the manufacturer indications. After transformation of competent *E. coli* cells, the resulting colonies were selected and analysed by colony PCR using the universal primers of the vector (M13 forward and T3 or alternatively M13 reverse and T7). Positive colonies were sent to sequence (Eurofins MWG Operon, Germany).

Information regarding primers and PCR parameters used will be provided upon request.

### RNA extraction and cDNA synthesis

Total RNA was isolated using NucleoSpin RNA II kit (Macherey-Nagel) according to the manufacturer’s instructions. Total amount of RNA was quantified by optical density (OD) using a Nanodrop 2000 Spectrophotometer (Thermo Scientific) and the purity was evaluated by measuring the ratio of OD at 260 and 280 nm. 1 μg of DNAse-I treated RNA was converted to cDNA by iScript cDNA synthesis kit (BioRad) following the manufacturer’s instructions and stored at −20 °C. A portion of the RT reaction (1/20 volume) was used to amplify the various genes with specific primer sets.

### Quantitative real-time polymerase chain reaction (qRT-PCR)

Quantitative PCR was performed in a thermocycler (StepOne Plus Real Time PCR System, Applied Biosystems) using the SSO Advanced Universal SYBR Green PCR Supermix (Biorad). The primers for the target human gene (ATXN3, NM_004993 and BECN1, NM_003766 were pre-designed and validated by QIAGEN (QuantiTect Primers, QIAGEN) and the reference gene for GAPDH was designed and validated in the lab:

F-TGTTCGACAGTCAGCCGCATCTTC

R-CAGAGTTAAAAGCAGCCCTGGTGAC

A master mix was prepared for each primer set containing the appropriate volume of SSO Advanced Universal SYBR Green PCR Supermix (Biorad), primers and template cDNA. All reactions were performed in duplicate and according to the manufacturer’s recommendations: 95 °C for 30 sec, followed by 45 cycles at 95 °C for 5 sec, 58 °C for 15 sec and 0.5 °C increment for starting at 65 °C at each 5 sec/step up to 95 °C. The amplification efficiency for each primer pair and the threshold values for threshold cycle determination (Ct) were determined automatically by the StepOne Software v2.3 (Applied Biosystems). The mRNA fold change with respect to control samples was determined by the Pfaffl method, taking into consideration different amplification efficiencies of all genes.

### Statistical analysis

Statistical computations were performed using GraphPad Prism version 5.0, GraphPad Software, La Jolla, CA, USA, www.graphpad.com. Statistical significance between groups was determined by unpaired Student t-test or one-way ANOVA for multiple comparisons, followed by Bonferroni test for selected pairs comparison. P-values < 0.05 were considered as statistically significant; *p* < 0.01 very significant; and *p* < 0.001 extremely significant.

## Results

### Establishment and characterization of primary human skin fibroblast cultures

**Figure 1:**
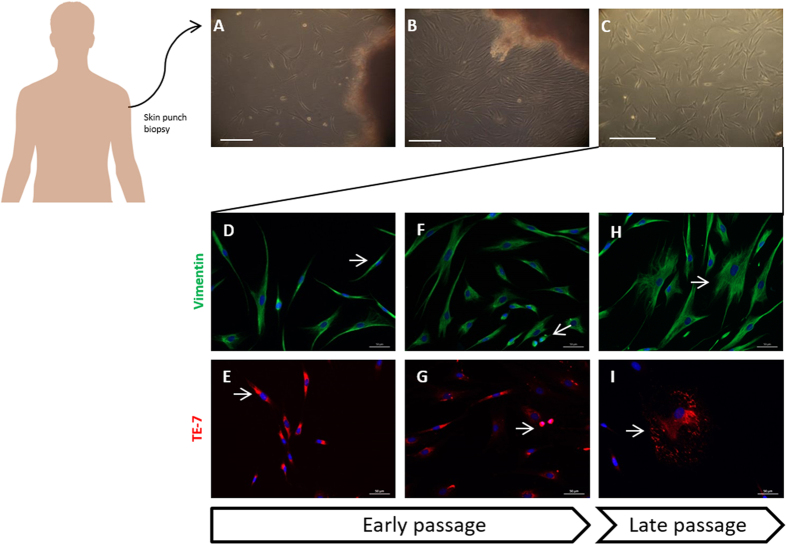
Fibroblasts primary cultures were derived directly from excised skin as explants. Fibroblasts cultures started to grow from the minced fragments in 5 days (**A**). After a week the cells reached confluency (**B**) and were detached enzymatically and plated for further expansion on passage P1 (**C**). Scale 250 μm. Immunostaining of fibroblasts with vimentin (green), TE-7 (red) and DAPI (blue). Starting cultures of fibroblasts and early passages were mainly composed/enriched in fusiform cells and bright cells, capable of division, (**D–G**) late passages instead display senescent cells in star-like shape (F,I).

Skin explants were obtained from healthy individuals and MJD patients followed at the Coimbra University Hospital Centre and cultured as previously described^15^,^16^,^20^ taking advantage of the outgrowing property of fibroblasts from skin, which enabled a high cell yield in a short period of time. Five days post-cultured fibroblasts presented the characteristic spindle-shape morphology, with elongated cell bodies, single oval nucleus and linear alignment of cellular distribution as previously described^21^ (Fig. 1A). Migration of fibroblasts from the cultured skin explant and cellularity were similar for both MJD and healthy control samples and no correlation was found with donor age^22^.

Once confluent (Fig. 1B), fibroblast cultures surrounding the pieces of skin were further expanded (Fig. 1C) and observed under vimentin and TE-7 immunostaining. All the cells were positive for vimentin, a common marker for mesodermal-derived tissues as dermis and for TE-7, a specific marker for fibroblasts^23^. Together these results confirm the purity of the established fibroblast cultures.

Early passages of fibroblasts were mainly composed by fusiform (Fig. 1D,E, arrows) and dividing cells (Fig. 1F,G, arrows); in contrast, late passages were progressively enriched in non-dividing star-like shaped senescent cells (Fig. 1H,I, arrows) as a result of replicative senescence, regardless of the genotype, gender and age of the donor.

**Figure 2:**
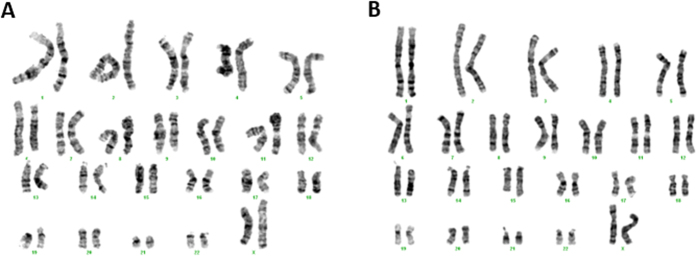
Chromosome analysis of fibroblast primary cultures. Representative aligned karyotype of CTRL and MJD primary fibroblasts (**A** and **B**, respectively). The results revealed a normal diploid karyotype of 2n = 46 for all the established cultures of primary fibroblasts.

In agreement with previous reports^24^, fibroblast cultures presented a normal diploid karyotype with no aberrant modifications (Fig. 2).

We further performed genetic characterization through DNA sequencing for the *ATXN3* gene, and we found that normal non-expanded CAG repeats in control and MJD fibroblast cultures varied similarly from 14 to 23, whereas the expanded allele ranged from 70 to 80 (Table 1).

Interestingly, the subjects exhibiting a severe phenotype of the disease, based on SARA scores and clinical evaluation, also presented the highest number of CAG repetitions reinforcing the correlation between CAG expansion size and disease severity previously described^25^. We also analyzed the exon and intron 10 of *ATXN3* gene for the presence of the 3 flanking single-nucleotide polymorphisms (SNPs) associated to the (CAG)_n_ region, namely C^987^GG/G^987^GG (SNP rs12895357), TAA^1118^/TAC^1118^ (SNP rs7158733) and C^1178^/A^1178^ (SNP rs3092822)^26^,^27^. We found the ACA and GGC haplotypes (related to Flores and São Miguel island, respectively) in the MJD patients in study.

### Assessment of ataxin-3 levels in human fibroblasts cultures

**Figure 3:**
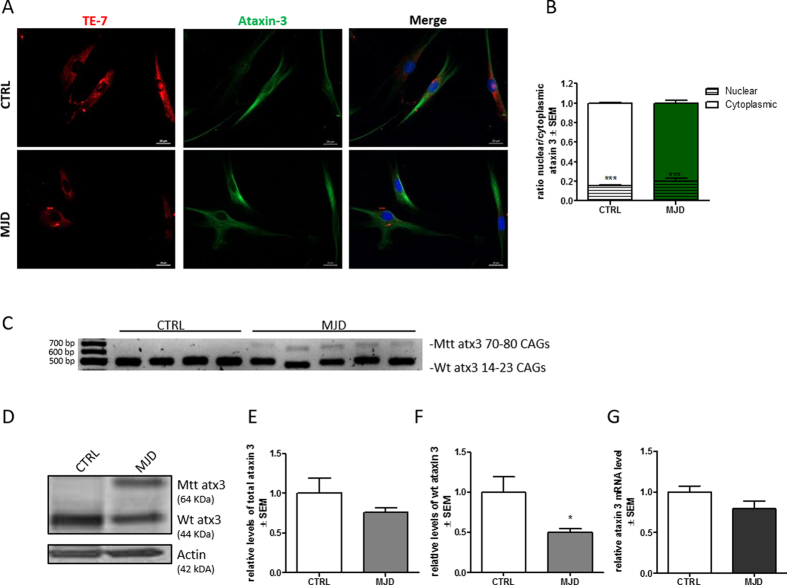
Ataxin-3 is predominantly a cytoplasmic protein for CTRL and MJD fibroblasts with similar total levels. Immunostaining of cultured fibroblasts obtained from skin biopsies with TE-7 (red), ataxin-3 (green), and DAPI (blue) (**A**). Quantification of nuclear and cytoplasmic ataxin-3, based on ataxin-3 immunoreactivity (**B**). Levels of nuclear ataxin-3 were significantly lower than cytoplasmic ones (Student t-test n = 3/n = 3 ***p = 0.001). RT-PCR analysis of transcripts from control and MJD fibroblast cultures (**C**). Representative western blot of wild-type (44kDa) and mutant ataxin-3 (64 KDa) in CTRL and MJD fibroblasts (**D**). Densitometric quantification of total ataxin 3 (p = 0.1725 Student t-test n = 3/n = 5) (**E**) and wild type ataxin 3 (**F**) (Student t-test n = 3/n = 5 *p = 0,05) relative to actin. qRT-PCR analysis of total level of ataxin-3 relative to GAPDH (p = 0.1833 Student t-test n = 3/n = 5) (**G**). Full length agarose gel and western blot membranes are provided in supplementary information, S1.

To clarify whether MJD would modify the subcellular localization and levels of ataxin-3 in control and patient fibroblasts we analyzed cultures by immunofluorescence and western blot. We found that ataxin-3 was predominantly located in the cytoplasm of cells, in granular and fibrillar form (Fig. 3A) as previously described^28^,^29^. The subcellular location of ataxin-3 was similar in control and MJD fibroblast cultures, with 16% ± 0.003 of nuclear ataxin-3 in control fibroblasts and 20% ± 0.019 in MJD fibroblasts. Ataxin-3 was located mainly in the cytoplasm and no aggregates or inclusions were found (Fig. 3B). The presence of ataxin-3 in a non-neuronal cell type as fibroblasts confirms the ubiquous expression of ataxin-3^30^.

Levels of wild-type and mutant ataxin-3 were then analyzed by RT-PCR (Fig. 3C) and western blot (Fig. 3D–F). All patient cells were derived from heterozygous MJD patients and therefore exhibited expression of both mutant and wild-type ataxin-3. As expected we found that the sum of wild-type and mutant ataxin-3 protein levels in MJD fibroblasts was similar to the levels of wild-type ataxin-3 in healthy controls (Fig. 3E) and that the levels of wild-type ataxin-3 in patient cells were half those found in controls. qPCR analysis of mRNA levels of ataxin-3 further confirmed that the levels of ataxin-3 were similar in both groups (Fig. 3G).

### Defining MJD autophagic dysfunction phenotype: autophagy is impaired in MJD fibroblasts

In order to investigate whether the MJD genotype was associated with a cellular dysfunction in patient fibroblasts we evaluated the levels of beclin-1, as previous described^6^ and two crucial autophagic flux related proteins: p62/SQSTM1 and LC3-II.

**Figure 4:**
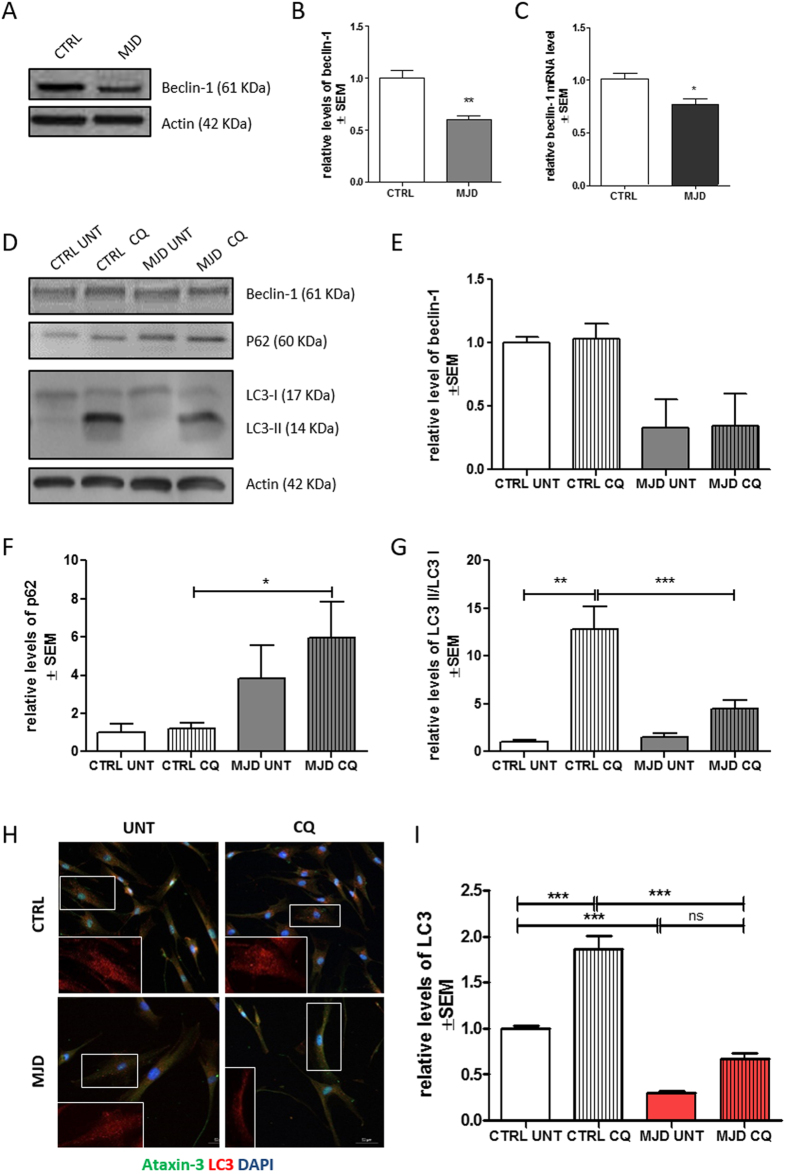
Defining MJD autophagic dysfunction phenotype. Representative western blot of Beclin-1 (61 kDa) (t student n = 3/n = 4 **p = 0.01) in human fibroblasts (**A,B**) and qRT-PCR analysis of beclin-1 (t student n = 3/n = 4 *p = 0.05 (**C**). Representative western blots (**D**) of beclin-1 (61 kDa) (**E**), p62 (60 kDa) (**F**), LC3-II and LC3-I (14 and 16 kDa) and LC3-II/LC3-I (**G**) levels in human fibroblasts after treatment with chloroquine (CQ). (**H**). 1way ANOVA with Bonferroni post test (*p < 0.05; **p < 0.01) n = 3/n = 3. Immunostaining of cultured fibroblasts obtained from skin biopsies with ataxin-3 (green), LC3 (red), and DAPI (blue) (H). Quantification of LC3, based on LC3 immunoreactivity, normalized for CTRL UNT condition (I). Levels of LC3 were significantly higher after chloroquine treatment in CTRL (One way ANOVA n = 3/n = 3***p = 0.001). Scale bar: 50 μm. Full length western blot membranes are provided in supplementary information, S2.

Protein and mRNA levels of beclin-1 were significantly decreased in MJD condition (Fig. 4A–C), which suggests an impairment on the early step of vesicle nucleation of autophagic pathway ^4^. We did not find a significant difference in protein levels of beclin-1, p62/SQSTM1 and LC3-II (Fig. 4D–F and G) in MJD group as compared with the control group in basal untreated conditions. Nevertheless, a tendency for abnormal accumulation of p62 and decrease of LC3 II was observed in basal autophagic flux state, as verified by comparing control and MJD untreated conditions (untreated conditions - UNT). This tendency was already suggestive of an autophagy defective phenotype^31^.

We next sought to further investigate the putative autophagic dysfunction related with the MJD genotype by treating both MJD and control fibroblasts with chloroquine, an autophagic inhibitor which prevents autophagosome-lysosome fusion and subsequent proteolysis by raising the lysosomal pH^31^,^32^,^33^. Levels of beclin-1 (Fig. 4E) remained similar for both control and MJD conditions, in presence or absence of chloroquine. On the contrary P62/SQSTM1, an autophagy substrate, presented significantly increased levels in MJD samples treated with chloroquine as compared to control cells under the same treatment (Fig. 4F). This accumulation of p62/SQSTM1 in MJD fibroblasts upon chloroquine treatment indicates an impairment in autophagic flux.

The levels of LC3-II, a protein found on both the lumenal and cytosolic surfaces of mature autophagosomes, and the conversion of LC3-I in LC3-II were also investigated for both MJD and control fibroblasts in the presence and absence of chloroquine, as a way to analyze the autophagic flux dynamics in the presence of a blocker of fusion of autophagosomes with the lysosomes. In the chloroquine condition, MJD fibroblasts presented drastically reduced levels of LC3-II/LC3-I when compared with control fibroblasts suggesting incapacity to properly activate autophagy (Fig. 4D,G). These results indicate that the reduction of LC3-II levels is due to an impaired generation of autophagosomes. We further confirmed by immunostaining for LC3 that the levels of LC3 are abnormally decreased in MJD fibroblasts (Fig. H,I).

**Figure 5:**
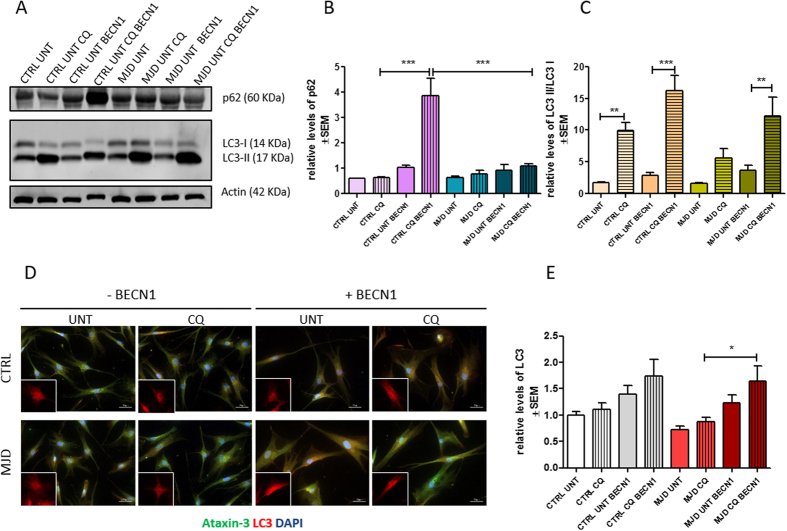
Overexpression of beclin-1 in MJD fibroblasts reverts MJD autophagic dysfunction phenotype. Representative western blots (**A**) of p62 (60 kDa) (**B**) and LC3-II and LC3-I and respective quantification of p62 (**B**) and LC3-II/LC3-I levels relative to actin (**C**) of human fibroblasts transduced with beclin-1 (BECN1) in the presence (CQ) or absence (UNT) of chloroquine; 1way ANOVA with Bonferroni post test (**p < 0.01, ***p < 0.001) n = 1/n = 3. Immunostaining of fibroblasts with ataxin-3 (green), LC3 (red), and DAPI (blue) (**D**). Quantification of LC3, based on LC3 immunoreactivity, normalized for CTRL UNT condition. Levels of LC3 were significantly higher after chloroquine treatment in MJD fibroblasts overexpressing beclin-1 (**E**); One way ANOVA with Bonferroni post test (*p < 0.05) n = 1/n = 3. Full length western blot membranes are provided in supplementary information, S3.

We then investigated whether the overexpression of beclin-1 could activate autophagy and revert the phenotype of autophagy impairment. We overexpressed beclin-1 in fibroblasts by transduction with a previously described^17^ lentiviral vector encoding for human beclin-1. Our results show that in spite of the activation of autophagy by means of beclin-1 overexpression, the levels of p62 were not restored in transduced MJD fibroblasts (Fig. 5A,B) as compared with the same condition in CTRL fibroblasts. Nevertheless, upon beclin-1 overexpression the ratio of LC3-II/LC3-I in MJD fibroblasts was found to be similar to CTRL fibroblasts (Fig. 5C). Overall these results suggest that the autophagy impairment found in fibroblasts is related with a decreased number of autophagosomes, as the levels of p62 are low even when autophagy is activated by beclin-1 overexpression. On the other hand, the ratio of LC3-II/LC3-I and LC3 total levels (Fig. 5C,D) are enhanced in MJD fibroblasts overexpressing beclin-1, particularly in the presence of chloroquine, reaching similar levels as CTRL fibroblasts (Fig. 5E), which indicates that although the number of autophagosomes is decreased, the autophagic flux can be restored.

Together these results indicate that autophagy is impaired in MJD fibroblasts and suggest that the underlying mechanism is the reduced synthesis of autophagosomes.

## Discussion

In this work we found an autophagy impairment phenotype associated with MJD in dermal fibroblasts obtained from patient’s biopsies. This phenotype is MJD specific and a consequence of defective generation of autophagosomes that can be partially reverted by beclin-1 overexpression.

We established MJD human primary cultures of fibroblasts, with preserved diploid karyotype characteristic from the tissue of origin (Fig. 2) and we provided its characterization in terms of disease genotype (Table 1) and autophagy related phenotype. The standardization of the conditions for explantation and subcultivation of the skin fibroblast cultures were observed^34^, as both MJD and control groups of skin fibroblast cultures presented similar characteristics regarding cell growth, cellularity and normal karyotype (Figs 1 and 2). Interestingly, we found the ACA haplotype^35^ in patients with most severe clinical outcome, as measured by SARA score (Table 1) and we also found for these patients a correlation between increased CAG expansion size and earlier age of onset, as previously described^36^. The C variant for the mutant allele is the most common^37^,^38^ and it is a distinctive feature that allows the use of allele-specific siRNA silencing^39^,^40^ and other gene editing strategies in neuronal *in vitro* models derived from MJD fibroblast, as therapeutic strategies for MJD.

Distribution and levels of mutant ataxin-3 are important for the manifestation of the neurodegenerative phenotype and the presence of protein aggregates in the nucleus of neuronal cells in the form of NIIs (neuronal ubiquitinated intranuclear inclusion bodies) are considered a hallmark of MJD^1^,^41^,^42^,^43^. In our study, we found that ataxin-3 distributed predominantly in the cytoplasm of the fibroblasts as described previously for both neural and non-neural tissues^28^. In MJD fibroblasts, ataxin-3 was also found in the nucleus (Fig. 3A), but not in the ubiquitinated form (data not shown) as reported for neurons targeted by the disease^1^,^28^. We found similar levels of total ataxin-3 for both control and MJD fibroblasts (Fig. 3D,E), but as expected the levels of wild-type ataxin-3 in MJD fibroblasts were decreased by 50% (Fig. 3F).

It has been reported that reduced autophagy induction, altered cargo recognition, inefficient autophagosome/lysosome fusion or inefficient degradation of the autophagic cargo in lysosomes were potential defects underlying autophagy malfunction in neurodegenerative diseases^4^,^44^,^45^,^46^. In this study, we addressed the general cellular effects of mutant ataxin-3 on autophagic flux, using three different markers to qualify the underlying malfunction: beclin-1 levels, p62/SQSTM1degradation^47^ and the levels of LC3-II^10^. Cellular levels of beclin-1 are often correlated with autophagic activity as the reduced expression in neurodegenerative diseases is linked to autophagy impairment^48^. Also the accumulation of p62/SQSTM1 is a reliable indicator of autophagy suppression when used in combination with LC3-II turnover^31^, a marker closely correlated with the number of autophagosomes and thus with autophagosome formation.

We found reduced levels of beclin-1 in MJD fibroblasts (Fig. 4A–C), as described before^6^. In order to estimate the dynamics of autophagic flux in MJD fibroblasts and to confirm the autophagy-defective phenotype, we added a new study condition, by blocking fusion of autophagosomes with lysosomes with chloroquine. We found unaltered levels of beclin-1 after chloroquine treatment in both control and MJD fibroblasts (Fig. 4E) which was expected, given that this protein is not a substrate in the autophagic pathway and rather plays a role in its early step of autophagy initiation^49^. On the contrary, P62/ SQSTM1 was accumulated in chloroquine condition. Having in mind that p62 is a substrate degraded during the course of autophagic flux, this accumulation corroborates the indication of autophagy impairment (Fig. 4F). Simultaneouslly, in control fibroblasts LC3-II levels were increased due to the expected autophagosome accumulation in a situation of uncompromised autophagic flux. Importantly, in MJD fibroblasts the defective autophagic machinery was unable to produce the expected increase in LC3-II levels observed in control fibroblasts.

These results indicate that the autophagic flux is compromised in MJD fibroblasts based on data from basal and dynamic autophagic flux. Moreover, our results suggest that the possible underlying mechanism is the reduced autophagosome synthesis based on LC3-II and LC3 levels (Fig. 4G,I).

Overexpression of beclin-1 in MJD fibroblasts restored the autophagic flux to similar levels of the ratios of LC3-II/LC3-I found in CTRL fibroblasts (Fig. 5B,E). Nevertheless, the levels of p62 remained low (Fig. 5B) even after autophagy activation suggesting that other mechanisms involved in the regulation of autophagosome formation besides the one activated by beclin-1, such as the mTOR signaling pathway, are impaired and responsible for the observed phenotype of reduced autophagosome synthesis^50^.

Altogether our results suggest that fibroblasts can be used as a MJD *in vitro* model for initial tests of drug screening targeting autophagy impairments and gene repair therapies that can be applied in *in vitro* neuronal models, speeding up clinical translation.

## Additional Information

**How to cite this article**: Onofre, I. *et al*. Fibroblasts of Machado Joseph Disease patients reveal autophagy impairment. *Sci. Rep.* **6**, 28220; doi: 10.1038/srep28220 (2016).

## Supplementary Material

Supplementary Information

## Figures and Tables

**Table 1:**
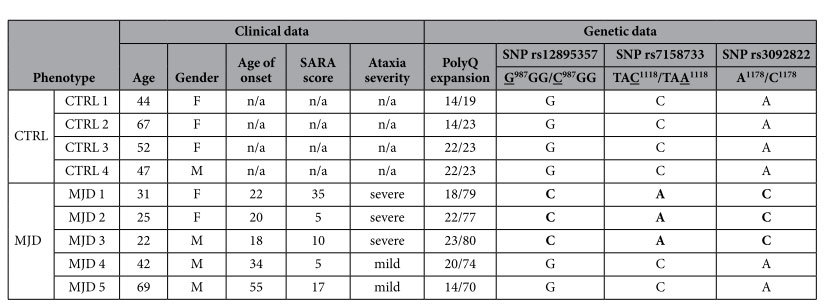
Clinical and genetic data of healthy individuals and MJD patients included in the study.

Fibroblasts were obtained from five MJD patients who came from four families (patients MJD 2 and MJD 3 are siblings) and healthy individuals, with no history of neurological disease, related (CTRL 3 is the non-affected sister of MJD1) or not related with MJD patients. The ataxia severity was evaluated based on symptoms and scores on the Scale for the Assessment and Rating of Ataxia (SARA, range: 0 – 40). Ratios above or equal to 1 were deemed “severe” and ratios of < 1 was deemed “mild”. n/a-not applicable.
