# Supplementary material for: Fibroblasts of Machado Joseph Disease patients reveal autophagy impairment
Source: Sci Rep. 2016 Jun 22;6:28220. doi: 10.1038/srep28220 (PMC4916410; doi:10.1038/srep28220)
Supplement: Supplementary Information [file srep28220-s1.pdf]

Fibroblasts of Machado Joseph Disease patients reveal autophagy impairment

Isabel Onofre[1,2¶], Nuno Mendonça[1,3,4¶], Sara Lopes[1,5], Rui Nobre[1,5], Joana Barbosa de Melo[,1,4,6], Isabel Marques Carreira[1,4,6] Cristina Januário[3,4] António Freire Gonçalves[3,4] and Luis Pereira de Almeida[1,2]

1- CNC - Center for Neuroscience and Cell Biology, Coimbra, Portugal

2- Faculty of Pharmacy, University of Coimbra, Coimbra, Portugal

3- Neurology Department, Coimbra University Hospital Center, Coimbra, Portugal

4- Faculty of Medicine, University of Coimbra, Portugal

5- IIIUC- Institute for Interdisciplinary Research, University of Coimbra, Coimbra, Portugal

6-*Cytogenetics* and Genomics *Laboratory, Faculty of Medicine, University of Coimbra, Coimbra, Portugal*

¶ - Equal contribution

Corresponding author:

Luis Pereira de Almeida (luispa@cnc.uc.pt)

Suplemental Information

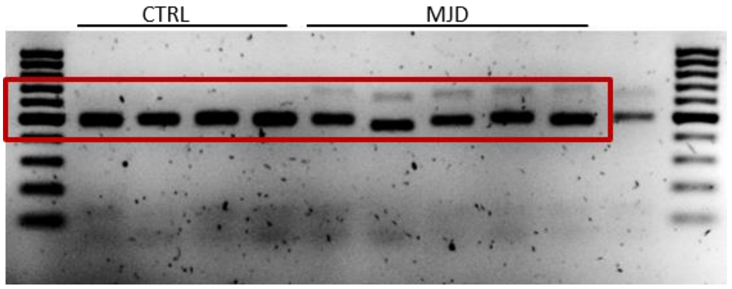

Agarose gel
Ataxin-3

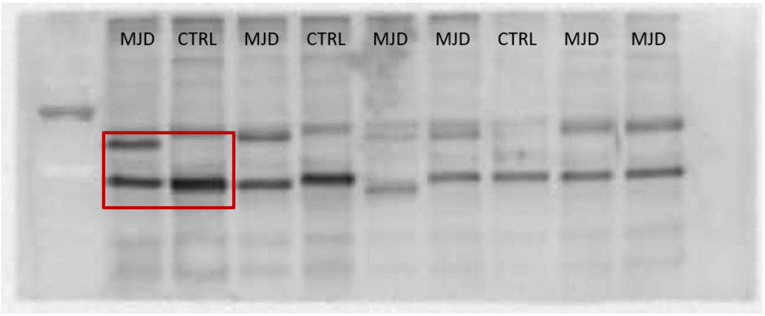

Western blot
membrane
Ataxin-3

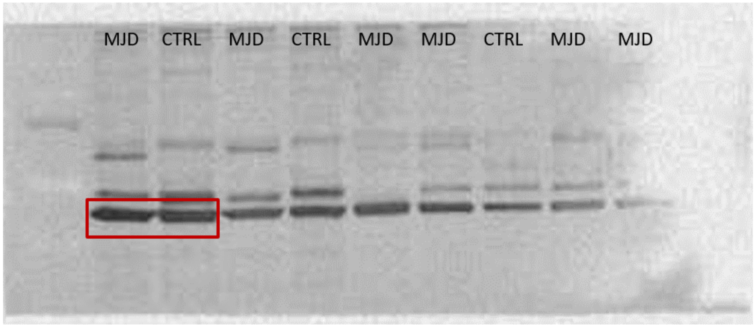

Western blot membrane
Actin/ Ataxin-3

S1- Full lenght agarose gel relative to Fig 3C and full length western blot membrane relative to Fig 3D. Red boxes indicate the cropping lines used to generate the figures.

Suplemental Information

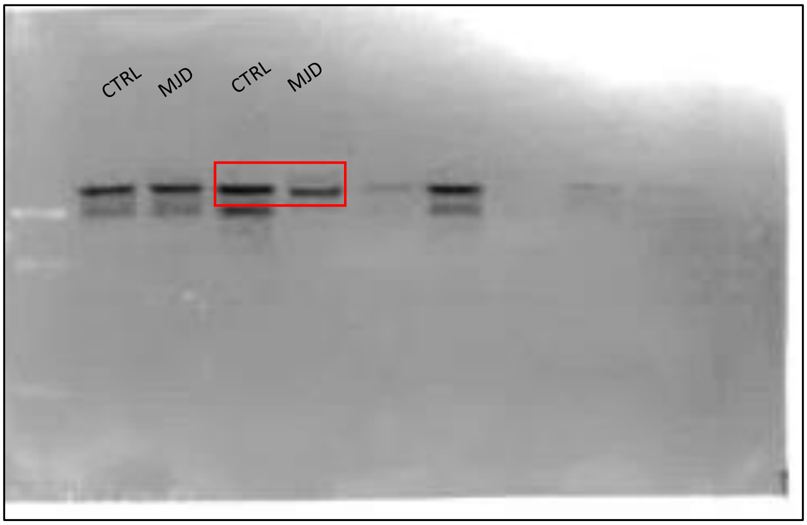

Western blot membrane Beclin-1

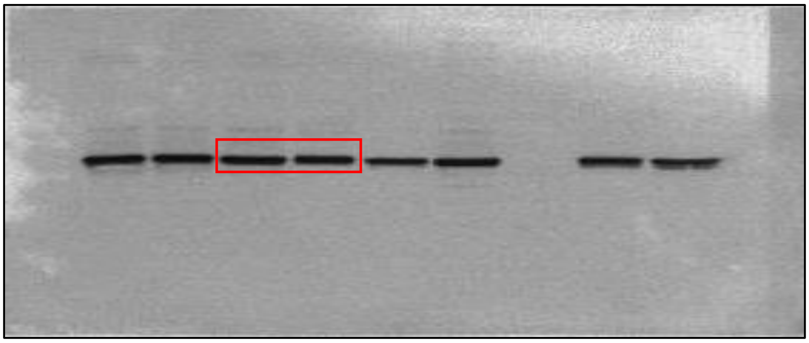

Western blot membrane Actin/Beclin-1

S2- Full lenght western blot membranes relative to Fig 4A. Red boxes indicate the cropping lines used to generate the figures.

Suplemental Information- Figure 4

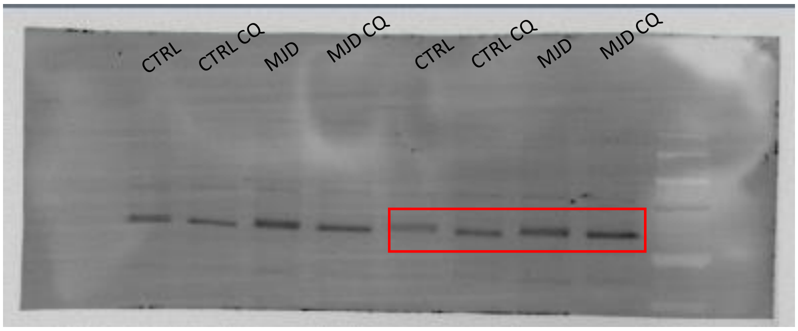

Western blot membrane p62

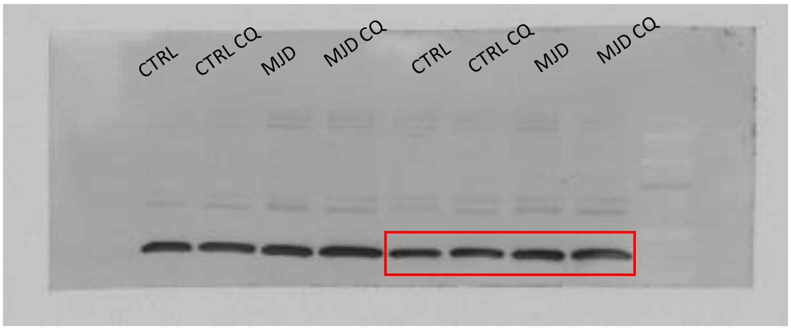

Western blot membrane Actin/p62

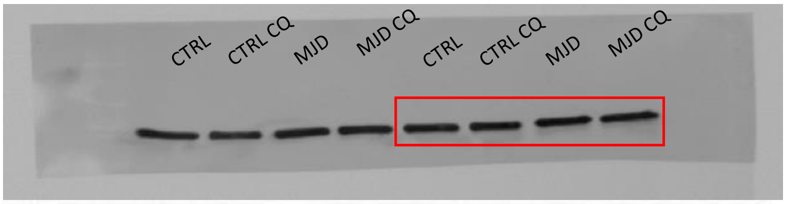

Western blot membrane Actin/LC3

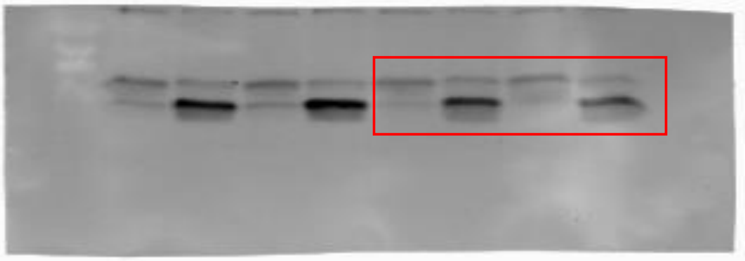

Western blot membrane LC3

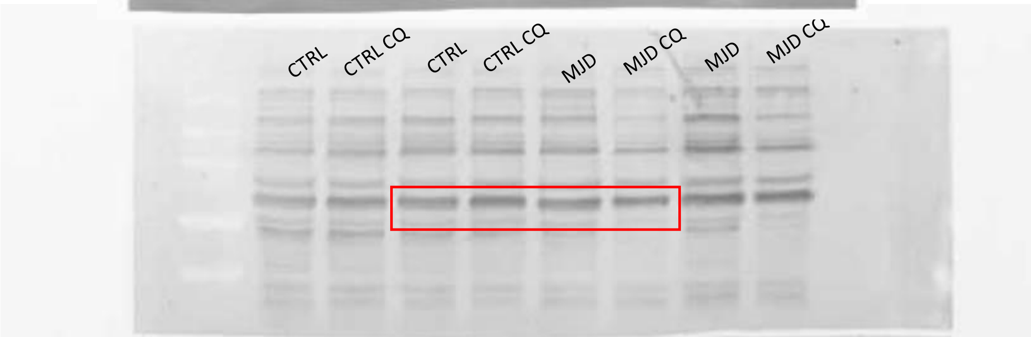

Western blot membrane Beclin-1

S2- Full lenght western blot membranes relative to Fig 4D. Red boxes indicate the cropping lines used to generate the figures.

A

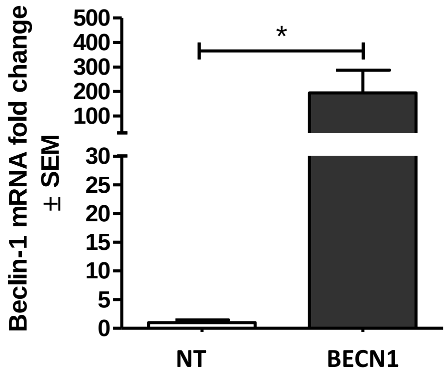

B

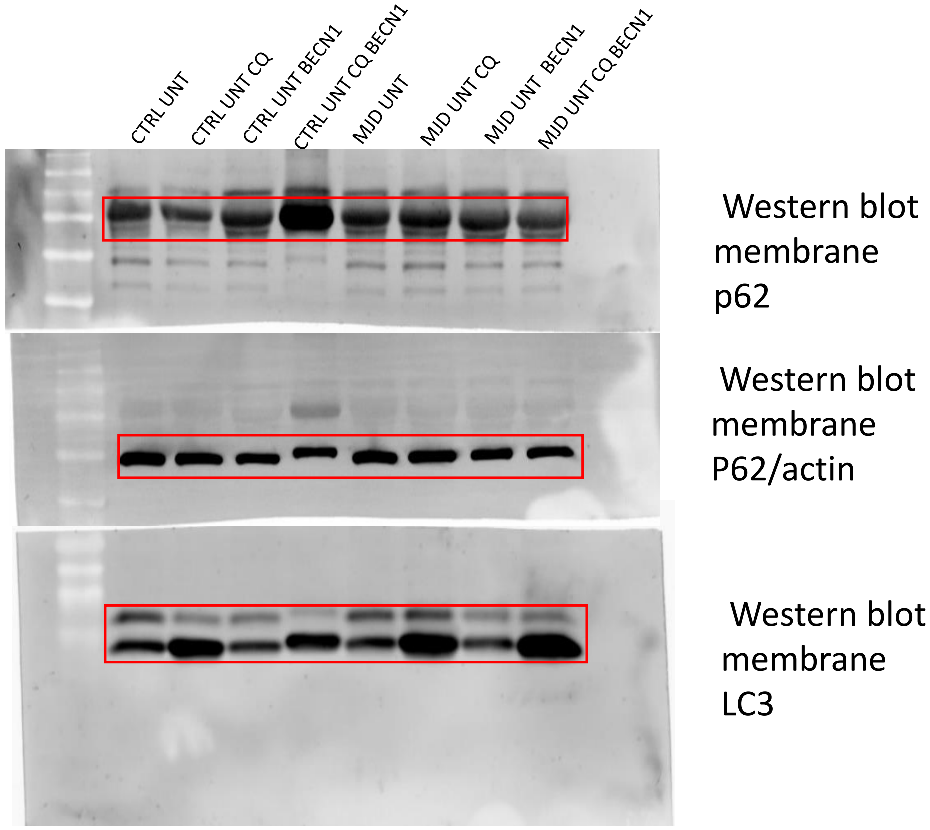

S3- A- Quantification of Beclin-1 mRNA levels on transduced fibroblasts after transduction with a lentiviral vector coding for human beclin-1. GAPDH was used as reference gene and presented levels were normalized for NT (non transduced) condition (Student t-test n=4/n=4 *p=0.05).

B-Full lenght western blot membranes relative to Fig 5A. Red boxes indicate the cropping lines used to generate the figures.
